# Product Biomonitoring and Responsible Reporting

**DOI:** 10.1289/ehp.1003355

**Published:** 2011-02

**Authors:** Paul Jung

**Affiliations:** Chief of Staff, NIEHS, National Institutes of Health, Department of Health and Human Services, Research Triangle Park, North Carolina, E-mail: jungp@niehs.nih.gov

In this issue of *Environmental Health Perspectives* (*EHP*), Schecter et al. (2011) report levels of polybrominated diphenyl ether (PBDE) flame retardants in butter purchased from retail stores. Although the investigators found prevalent levels of PBDEs in butter, they report that one sample had inordinately high levels of octa-, nona-, and deca-BDE congeners, likely from its highly contaminated wrapping paper.

The safety of PBDEs has come into question (DiGangi et al. 2010). Chemical companies have voluntarily phased out penta- and octa-BDEs in the United States and have agreed to do so for deca-BDE production, and some states have banned PBDE use in consumer products such as mattresses and electronics (U.S. Environmental Protection Agency 2007U.S. Environmental Protection Agency 2010). The presence of these chemicals in food products has not been addressed explicitly in legislation or regulations in the United States.

Given the lack of clear regulatory guidance and the question of risk from high levels of PBDEs in food products, it is legitimate to ask: should investigators in these types of “product biomonitoring” studies, when confronted with startling results from any potentially risky chemical, report their results to the manufacturer or to any regulatory agency prior to publication? Should the investigators identify specific brand name products and their manufacturers in their manuscript? More important, would the public want to know such results?

In a study of clinical trial subjects in brain imaging studies, Kirschen et al. (2006) found that at least 91% of the subjects, depending on the setting, wanted incidental findings to be disclosed to them, regardless of their clinical significance. In a workshop at which investigators discussed incidental findings, Illes (2006) found that most of the participants believed that research protocols should provide for disclosure of incidental findings. In environmental exposure studies there is no clear consensus on reporting individual data (Morello-Frosch et al. 2009), but Brody et al. (2007) found that an overwhelming majority of subjects (97%) were interested in knowing their personal results, regardless of the limited or absence of scientific information related to the health risks of that data.

But the study by Schecter et al. (2011) is not a clinical trial, nor does the study include human subjects with measurements of internal exposures. On the other hand, the butter obtained for this study would otherwise have been purchased and consumed; additional lots of butter, similarly contaminated, may be awaiting purchase or may have already been consumed. In cases such as this, do clinical trial and environmental exposure reporting recommendations apply, including any ethical requirements to notify subjects of incidental findings? And who are the “subjects” in such product biomonitoring studies?

A secondary question relates to the planning of such research: Should product biomonitoring protocols plan for reporting of results? Should this issue be addressed in a prestudy protocol, perhaps subject to vetting by an institutional review board (IRB)–like committee with data safety monitoring board (DSMB) oversight? Clearly, product biomonitoring studies without human subjects fall outside the realm of current IRB and DSMB jurisdiction. Given that IRBs and DSMBs are assembled to determine the risks to study participants, product contaminants of questionable risk may be too ambiguous for clear direction. These oversight bodies may thus be placed in a difficult position of interpreting the science behind a particular contaminant in addition to advising the conduct of a research study. Regardless, it would be appropriate for investigators who conduct such studies to explicitly identify in their protocol procedures for handling and reporting results, both expected and unexpected. This may have implications on where and how data will be submitted for publication and use, but will likely help guide the investigators when surprising, incidental findings arise. Hernick et al. (2011) recently described their experience communicating unexpected biomarker results in a population of girls; their model may provide guidance for future research endeavors.

Some people may fear that making product biomonitoring data public might generate alarm or panic among consumers, but research indicates this is unlikely (Altman et al. 2008). Schecter’s research team has already published data on PBDEs in composite food samples (Schecter et al. 2010a) and on bisphenol A in identified, brand-name food products (Schecter et al. 2010b) without apparent widespread panic among the public. In addition, the Food and Drug Administration (FDA) conducts regular market basket studies as part of its Total Diet Study (TDS) (FDA 2010). The TDS does not measure PBDEs (FDA 2009a), but it does measure other chemicals of concern in common consumer food products; results are made public (FDA 2009b), but the FDA does not identify specific brand names of sampled products or their manufacturers in their public data reports. The FDA does, however, pursue regulatory action if warranted by TDS results (Egan 2002).

Given the preferences of subjects in environmental exposure studies and the practice of the FDA in the TDS, it is clearly reasonable to make results of product biomonitoring studies public. But should a specific brand-name product or manufacturer be identified? One consideration is whether the product sampled in a study is representative of the totality of that product, or merely a small, inadvertently contaminated production lot. It would be unfair for a single sample to tarnish the reputation of a product without additional data. In addition, the specter of litigation looms over such decisions (Ledford 2008). Reporting of results tied to a specific product or manufacturer may result in an injunction against publication, accompanied by a subpoena for original data or litigation to repeat studies for validation purposes prior to release of results. The journal publishing the paper may also find itself a party to such litigation.

Schecter et al. (2011) indicate that they informed the butter manufacturer of their results. This was clearly a responsible act on their part. Making results public by publishing this important information in an open access journal such as *EHP* is also appropriate. Product manufacturers should act, of course, in the best interest of the public’s health. The issue of results notification in product biomonitoring studies is worthy of more detailed examination, and consensus among researchers and journals should be a priority.

## Figures and Tables

**Figure:**
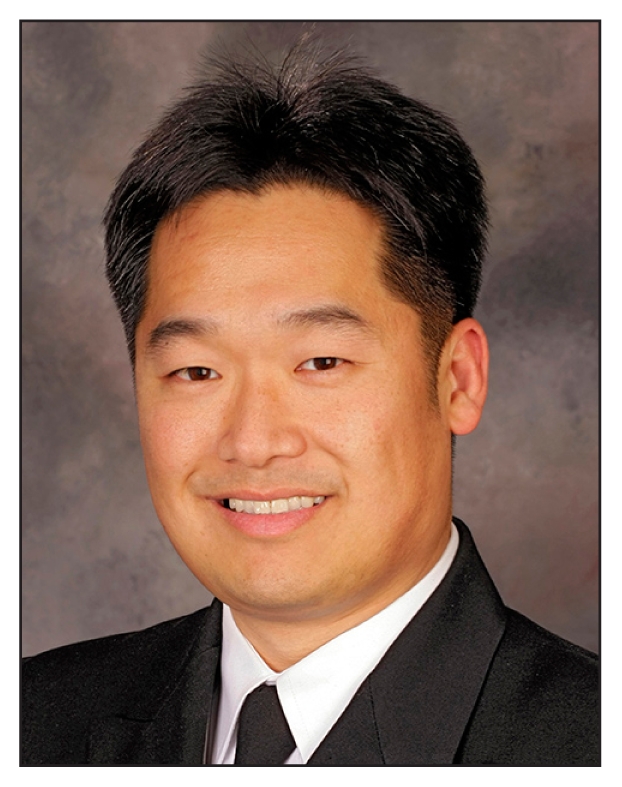
Paul Jung
